# Supplementary material for: Muscular expression of pezo-1 differentially contributes to swimming and crawling production in the nematode C. elegans
Source: bioRxiv. 2024 Aug 16:2024.08.13.607367. Preprint. [Version 1] doi: 10.1101/2024.08.13.607367 (PMC11343145; doi:10.1101/2024.08.13.607367)
Supplement: Supplement 3 [file NIHPP2024.08.13.607367v1-supplement-3.pdf]

bioRxiv preprint doi: https://doi.org/10.1101/2024.08.13.607367; this version posted August 16, 2024. The copyright holder for this preprint (which was not certified by peer review) is the author/funder, who has granted bioRxiv a license to display the preprint in perpetuity. It is made available under aCC-BY 4.0 International license.

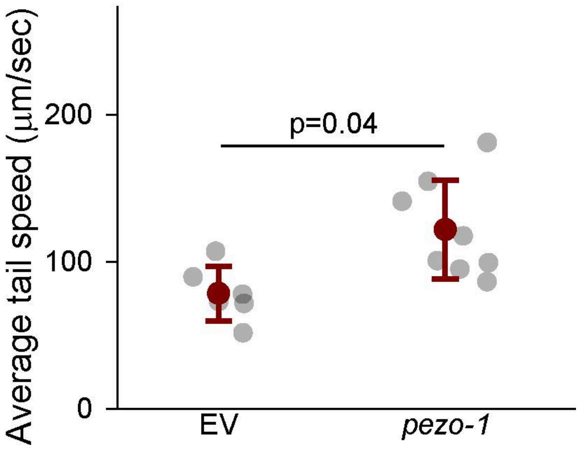

Figure S1. Increased crawling speed with *pezo-1* RNAi. RNAi targeting *pezo-1* expression increases the average speed of the tail during crawling compared to EV= **e**mpty RNAi **v**ector (L4440) used as control. Student t test. 1-β=0.49, Error bars = SEM. N>10 animals per each (datapoint) assay.

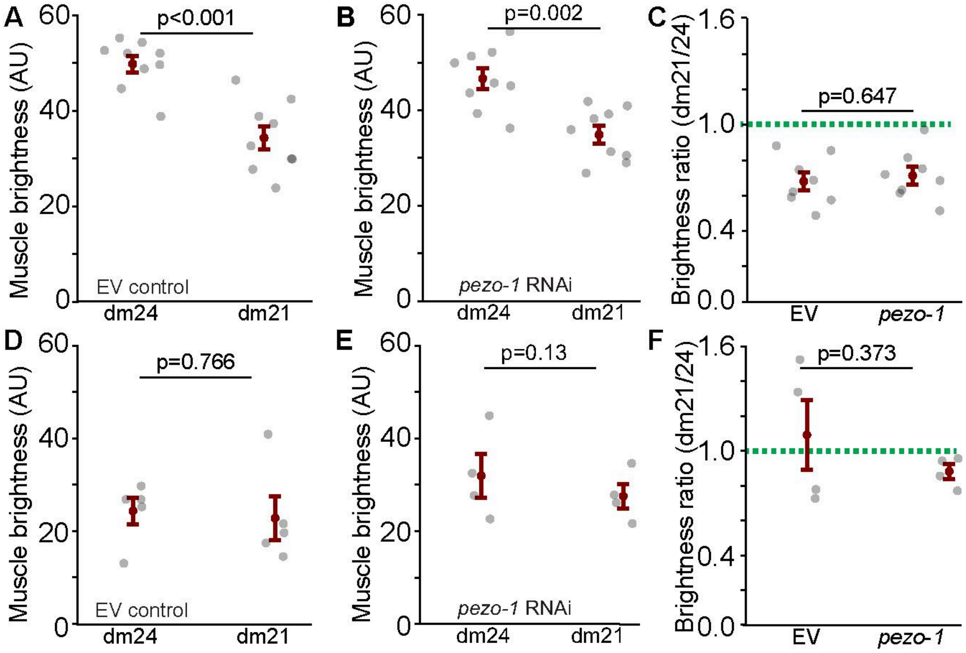

Figure S2. No selective increase in dorsal tail muscle activation during swimming with *pezo-1* RNAi. (A) Dorsal myocyte 21 and 24 activation during peak ventral and dorsal contraction in EV control animals during swimming. Paired t-test. 1-β=1.0. **(B)** Dorsal myocyte 21 and 24 activation during peak ventral and dorsal contraction in *pezo-1* RNAi treated animals during swimming. Paired t-test. 1-β=0.97. **(C)** Quantification shows no significant effect on relative activation of dm21 vs. dm24 with *pezo-1* RNAi during swimming peak tail bending. Welch's t-test. 1-β=0.83. N=10 animals. **(D)** Dorsal myocyte 21 and 24 activation during peak ventral and dorsal contraction in EV control animals during crawling. Paired t-test. 1-β=0.06. **(E)**

bioRxiv preprint doi: https://doi.org/10.1101/2024.08.13.607367; this version posted August 16, 2024. The copyright holder for this preprint (which was not certified by peer review) is the author/funder, who has granted bioRxiv a license to display the preprint in perpetuity. It is made available under aCC-BY 4.0 International license.

Dorsal myocyte 21 and 24 activation during peak ventral and dorsal contraction in *pezo-1* RNAi treated animals during crawling. Paired t-test. 1-β=0.3. **(F)**. Quantification shows no significant effect on relative activation of dm21 vs. dm24 with *pezo-1* RNAi during crawling peak tail bending. Welch's t-test. 1-β=0.89. N=5 animals. EV= **e**mpty RNAi **v**ector (L4440) used as control. Error bars = SEM.

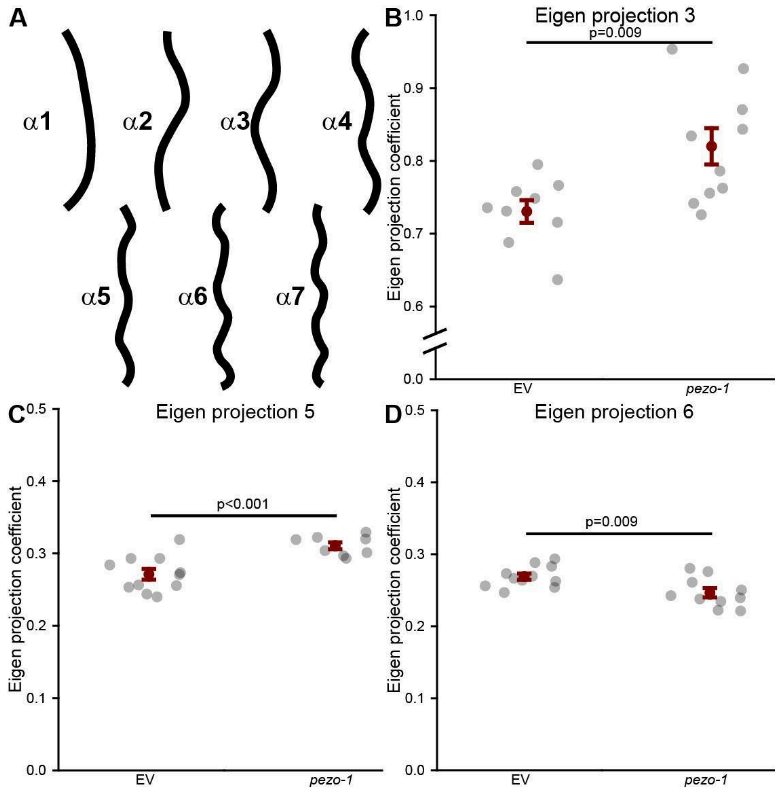

Figure S3. Alteration in swimming posture with *pezo-1* RNAi. A. Kinematic analysis performed using Tierpsy extracts the most common postural components of the animal behavior during swimming (eigen projections). RNAi targeting of *pezo-1* expression significantly impacted the eigen coefficients or projections 3, 5, and 6, resulting in a significant increase in contributions of projections 3 (**B**) and 5 (**C**), and a significant decrease in projection 6 (**D**). EV= **e**mpty RNAi **v**ector (L4440) used as control. Student's t-tests. 1-β>0.99. Error bars = SEM.
